# Postbiotics and Kidney Disease

**DOI:** 10.3390/toxins14090623

**Published:** 2022-09-06

**Authors:** Chiara Favero, Laura Giordano, Silvia Maria Mihaila, Rosalinde Masereeuw, Alberto Ortiz, Maria Dolores Sanchez-Niño

**Affiliations:** 1 Department of Nephrology and Hypertension, IIS-Fundacion Jimenez Diaz UAM, 28049 Madrid, Spain; 2 Division of Pharmacology, Utrecht Institute for Pharmaceutical Sciences, Utrecht University, 3584 CG Utrecht, The Netherlands; 3 Redes de Investigación Cooperativa Orientadas a Resultados en Salud (RICORS) 2040, 28049 Madrid, Spain; 4 Departamento de Medicina, Facultad de Medicina, Universidad Autónoma de Madrid, 28049 Madrid, Spain; 5 Departamento de Farmacología, Facultad de Medicina, Universidad Autónoma de Madrid, 28049 Madrid, Spain

**Keywords:** chronic kidney disease, postbiotics, prebiotics, probiotics, hyperoxaluria, *Oxalobacter formigenes*, GABA-salt

## Abstract

Chronic kidney disease (CKD) is projected to become the fifth global cause of death by 2040 as a result of key shortcomings in the current methods available to diagnose and treat kidney diseases. In this regard, the novel holobiont concept, used to describe an individual host and its microbial community, may pave the way towards a better understanding of kidney disease pathogenesis and progression. Microbiota-modulating or -derived interventions include probiotics, prebiotics, synbiotics and postbiotics. As of 2019, the concept of postbiotics was updated by the International Scientific Association of Probiotics and Prebiotics (ISAPP) to refer to preparations of inanimate microorganisms and/or their components that confer a health benefit to the host. By explicitly excluding purified metabolites without a cellular biomass, any literature making use of such term is potentially rendered obsolete. We now review the revised concept of postbiotics concerning their potential clinical applications and research in kidney disease, by discussing in detail several formulations that are undergoing preclinical development such as GABA-salt for diet-induced hypertension and kidney injury, sonicated *Lactobacillus paracasei* in high fat diet-induced kidney injury, GABA-salt, lacto-GABA-salt and postbiotic-GABA-salt in acute kidney injury, and *O. formigenes* lysates for hyperoxaluria. Furthermore, we provide a roadmap for postbiotics research in kidney disease to expedite clinical translation.

## 1. The Global Burden of Kidney Disease

Chronic kidney disease (CKD) is to date defined by the measurement of the estimated glomerular filtration rate (eGFR, i.e., a measure of kidney function) and albuminuria (i.e., a measure of kidney injury), which are associated with a combined risk of premature all-cause and cardiovascular death, CKD progression and acute kidney injury (AKI) [1]. Despite recent advances in the field, CKD is estimated to become the fifth global cause of death by 2040 and the second cause of death by the end of this century in countries with a long-life expectancy [2]. These projections illustrate key shortcomings in the current methods used to diagnose and treat kidney diseases. As such, an improved understanding of the underlying pathogenesis of CKD may help us identifying novel diagnostic clues and therapeutic targets. In this regard, the holobiont concept, a term used to describe an individual host and its microbial community, including viruses and cellular microorganisms [3], may pave the way to a better understanding of the pathogenesis of CKD. There is indeed increasing evidence suggesting that health and disease may be the result of a bilateral interaction between a host and its microbiota, particularly the gut microbiota. Such interaction may be affected by several factors: disease condition in the host, diet, drugs and antibiotics [4]. Attempts to modulate the host-microbiota interactions have resulted in the design and prescription of prebiotics, probiotics, synbiotics and postbiotics. As a result of a recent consensus on the definition of postbiotics [5], the pre-2019 literature on postbiotics and kidney disease has now become potentially obsolete. Accordingly, we here reviewed the concept and updated the literature on postbiotics in the field of kidney disease, by focusing on these interventions in line with the revised consensus on postbiotics concept.

## 2. The Gut Microbiota: A Key Modifier of Kidney Disease and Health

In recent years, a key role of the gut microbiota as a homeostasis modulator, influencing both health and disease, has emerged in the context of CKD [6,7]. Indeed, there is a close connection between intestinal dysbiosis, hypervolemia, systemic inflammation, myocardial stunning and the malnutrition-inflammation syndrome in CKD populations [8]. Kidney disease may influence the gut microbiota through several mechanisms, ranging from the influence of the uremic milieu to the impact of prescribed diets (e.g., low potassium diets are often low in dietary fiber), as well as the frequent use of antibiotics and polypharmacy which may adversely modulate the gut microbiota [9,10,11]. Conversely, an altered gut microbiota may contribute to CKD development and progression by mediating increased inflammation and/or generating uremic toxins and their precursors [12]. The increase in intestinal permeability resulting from the degradation of the barrier integrity, as a consequence of an altered microbiota, allows for the transition of endotoxins and bacterial products to the blood. This potentially leads to an increase in oxidative stress and inflammation, contributing to CKD complications, such as cardiovascular disease and mineral metabolism disorders. The gut microbiota may also generate indoles, amines and phenols from dietary components, mainly proteins. These gut-derived metabolites can be metabolized further into the uremic toxins p-cresyl sulphate (pCS), indoxyl sulphate (IS) and trimethylamine N-Oxide (TMAO) [9,11,13]. The presence of uremic toxins in the circulation may lead to proinflammatory events, promoting the expression of transforming factor-β (TGF-β1) and the production of reactive oxygen species (ROS) in kidney tubular epithelial cells [14]. IS and pCS also inhibit Klotho expression via gene hypermethylation [15]. Klotho has antiaging and nephroprotective effect. All these factors can lead to an acceleration of CKD progression and of its associated cardiovascular disorders. The recognition of the interplay between the kidneys and the microbiota has led to increased efforts in the development of novel therapeutic approaches focused on targeting the microbiota.

## 3. Prebiotics, Probiotics, Synbiotics and Postbiotics

**Figure 1:**
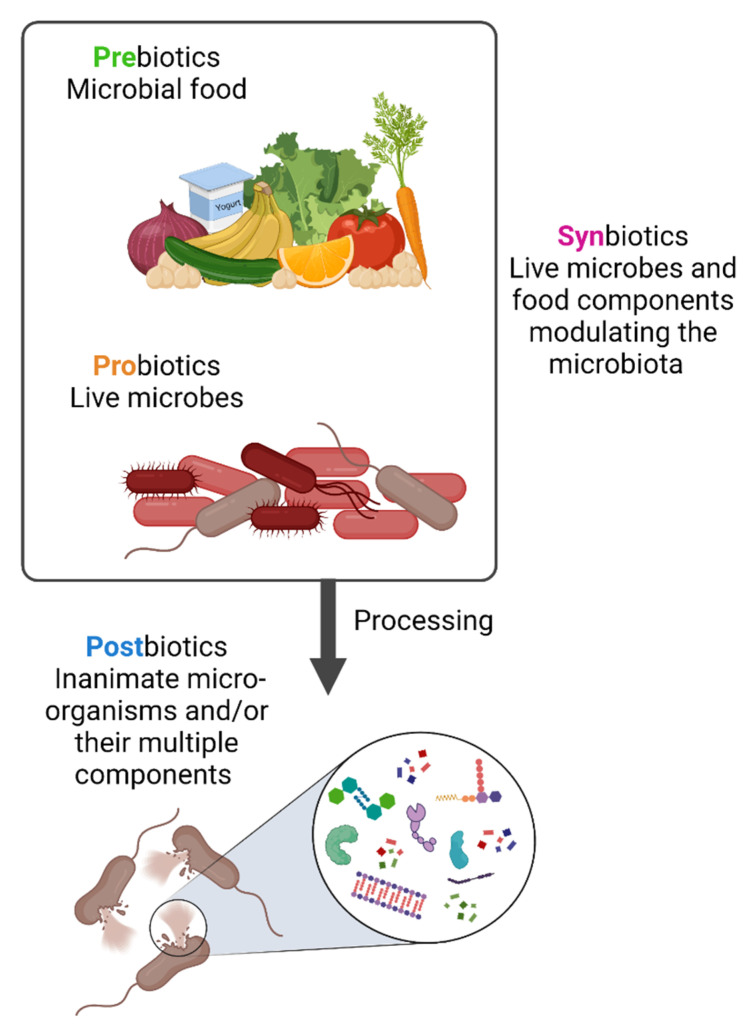
**Prebiotics, probiotics, synbiotics and postbiotics.** Prebiotics are molecules that can be metabolized by the microbiota while probiotics are live strains microorganisms, i.e., individual live components of the microbiota. When prebiotics and probiotics are used in combination these are named synbiotics. In contrast, postbiotics are the result of specific bacterial inactivation procedures. Figure created with Biorender.com (license number 9E1540F2).

Diet has a large impact on the composition of the microbiota as both humans and their gut microbiota use dietary components as nutrients. As a result, microbiota-modulating or -derived interventions have emerged, including probiotics, prebiotics, synbiotics and postbiotics, that could be available as drugs, diet or food supplements [16,17] (Figure 1). The concepts of probiotics, prebiotics and synbiotics have all been well described in the scientific literature.

The term “probiotic” is of Greek origin and means “for life”. It was probably first used in 1954 although its meaning has changed over time. The current definition was formulated in 2002 by the Food and Agriculture Organization of United Nations (FAO) and World Health Organization (WHO): “live strains of strictly selected microorganisms which, when administered in adequate amounts, confer a health benefit on the host”. The definition was adopted by the International Scientific Association for Probiotics and Prebiotics (ISAPP) in 2013 [18,19]. Prebiotics were defined in 2007 by FAO/WHO as “nonviable food component that confers a health benefit on the host associated with modulation of the microbiota”. Prebiotics are molecules that can be metabolized by bacteria in the gastrointestinal tract and include dietary fibers that are metabolized by bacterial enzymes to produce beneficial metabolites such as short chain fatty acids (SCFAs), including butyrate, acetate and propionate [20]. Prebiotics could be used either as an alternative to probiotics or combined with them [19]. In 1995, the term “synbiotic” was introduced to define the synergistic action of pro- and prebiotics. The main objective of synbiotics is to improve the survival rate of probiotics inside the intestinal tract, and an appropriate combination should provide an enhanced effectiveness in comparison to the use of each one individually.

Finally, another strategy able to promote health by reproducing the benefits of the gut microbiota is the use of postbiotics, an emerging microorganism-derived tool which we discuss in greater depth below.

## 4. The 2019 Concept of Postbiotic: What Is and What Is Not a Postbiotic

Although probiotics are live microorganisms, by the end of their shelf life they might get injured and die. Little attention has been paid to the contribution of these dead microorganisms and of the products of fermentation with regards to any biological impact of probiotics [5]. In 2019, the International Scientific Association of Probiotics and Prebiotics (ISAPP) defined the concept of postbiotic from a scientific, commercial, and regulatory point of view as a “preparation of inanimate microorganisms and/or their components that confers a health benefit on the host” [5]. “Preparation” indicates that it should have a specific formulation achieved through certain inactivation methods to confer any beneficial effects. “Inanimate” refers to the microorganisms that were alive and once they have been killed, they retain their beneficial effect. The word “components” focuses on the beneficial effect that might be played by microbial components, such as cell wall components and pili. While microbial metabolites might be present in postbiotic preparations and might play an essential role, the definition does not include the purified metabolites lacking cellular biomass. However, until 2019, the literature has frequently used the term incorrectly by referring to vaccines and purified metabolites, such as SCFAs, proteins and peptides as postbiotics [21,22,23]. This potentially renders obsolete any older literature using the term postbiotic and calls for an urgent reset.

The preparation of a postbiotic should follow defined criteria: firstly, the composition of the microorganism must be characterized prior to inactivation, for example through genome sequencing. Secondly, the process of inactivation should be described and verified. Thirdly, the postbiotic preparation must be reported [5].

The main advantages of postbiotics compared to probiotics lies in their stability. Their preparation and composition render them extremely stable for several years at room temperature, thus making them particularly suitable for those areas where the maintenance of the cold chain for transport and storage are still a limitation. In addition, they may have a better safety profile compared to probiotics as they cannot replicate and cause bacteremia or fungaemia. Nevertheless, safety should still be assessed for any kind of postbiotic [5].

**Figure 2:**
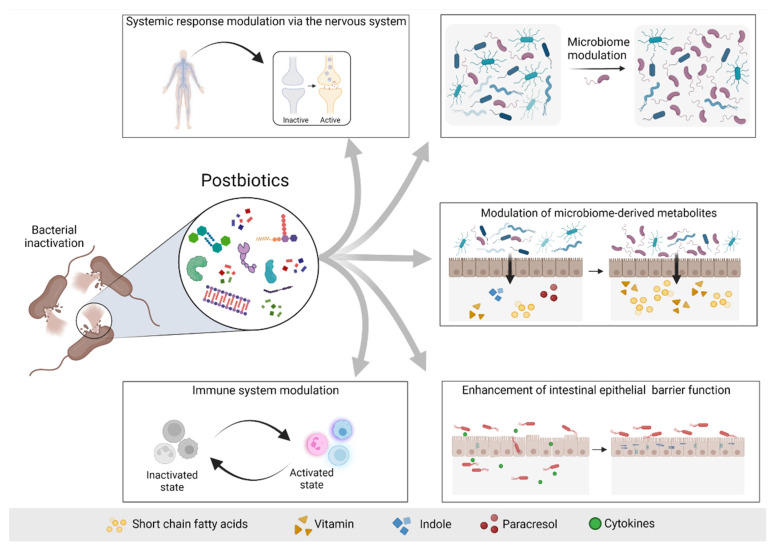
**Possible mechanisms of actions of postbiotics.** Postbiotics mediate their beneficial effects to the host via different mechanisms, including modulation of the systemic response via the nervous system (e.g., GABA), modulation of the microbiome and consequently modulation of the microbiome-derived metabolites, enhancement of the intestinal epithelial barrier and modulation of the immune system response. Based on [5]. Figure created with Biorender.com (license number 9E1540F2).

The possible mechanisms of actions of postbiotics include the modulation of resident microbiota, enhancement of epithelial barrier function, modulation of systemic or local immune responses, modulation of systemic metabolic responses and system signaling via the nervous system [5] (Figure 2).

## 5. Postbiotics in Non-Kidney Disease

Data from postbiotics in human studies are limited. Salminen et al. recently discussed the clinical postbiotic studies in adults and pediatric cohorts identified in the Cochrane central registration of controlled trials and in a MEDLINE database search for randomized controlled trials (RCTs), cohort studies and meta-analysis in adults and children [5] (Table 1 and Table 2) [24,25,26,27,28,29,30,31,32,33,34,35,36,37,38,39,40,41,42,43,44,45,46,47,48,49,50,51,52,53,54]. They identified fifteen clinical trials with postbiotics. Three studies tested postbiotics in gut diseases, these being two in irritable bowel syndrome (IBS) and one in chronic diarrhea. In five studies, postbiotics were used to treat pulmonary and respiratory diseases. The remaining others involved patients with cancer, obstructive jaundice, tuberculosis and helicobacter pylori. Three of them aimed to treat chronic stress or improve inflammatory response and performance during training [31]. Of these studies, eleven made use of inactivated bacteria and four of bacterial lysates.

Several studies reported efficacy for oral administration. Inactivated Lactobacillus acidophilus in Helicobacter pylori-positive patients treated with rabesprazole, clarithromycin and amoxicillin resulted in a higher eradication rate than antibiotics alone (*p* = 0.02) [24]. In patients with IBS, a heat-inactivated Bifidobacterium bifidum MIMBb75 decreased pain over the placebo group [26]. Patients with chronic diarrhea treated with heat-killed *L. acidophilus* LB (Lacteol Fort) also showed improved symptoms [27]. Medical students treated with heat-inactivated L. gasseri strain CP2305 showed a significant reduction in anxiety and sleep disturbance (*p* < 0.05) [30]. In pre-term infants, one RCT observed a reduced incidence in abdominal distention and lower fecal calprotectin (*p* = 0.001) when treated with formula fermented by Bifidobacterium breve and S. thermophilus [44]. A systematic review that considered four studies in healthy infants showed that fermented formula could provide benefits for gastrointestinal symptoms [55]. A meta-analysis of four RCT that involved children with acute gastroenteritis reported that heat-inactivated Lactobacillus acidophilus LB reduced the duration of diarrhea in hospitalized patients but not outpatients, compared to placebo [45,46,47]. In a postbiotic trial, the heat inactivated Lacticaseibacillus paracasei CBA L74 prevented common infectious diseases in children who were attending daycare probably by stimulating innate or acquired immunity [49]. Another clinical trial confirmed that supplementation with cow’s skim milk fermented with *L. paracasei CBA L74* could be a valid approach in preventing common infectious diseases in children [50]. Finally, one study investigated supplementation of infant formula with viable or heat-inactivated L. ramnosus GG and found that only viable L. ramnosus GG might be an efficient strategy to treat cow’s milk allergy and atopic eczema [52].

Overall, there is limited evidence suggesting that postbiotics may have beneficial effects in the treatment of diseases and this must be investigated in detail in well-designed controlled clinical trials.

## 6. Postbiotics in Kidney Disease

To our knowledge, there are no human studies conducted so far that investigated the use of postbiotics in kidney disease. However, a PubMed search performed in May 2022 identified several preclinical studies that examined the role and function of postbiotics in kidney-related diseases in animal models (Supplementary materials). In this search, we also found manuscripts published between 2020–2022 that used the term “postbiotic” to refer to compounds that would not be considered postbiotics according to the 2019 consensus definition [5]. In this regard, the short-chain fatty acid (SCFA) butyric acid and its derivative N-[2-(2-Butyrylamino-ethoxy)-ethyl]-butyramide (BA-NH-NH-BA) are produced by Cutibacterium acnes and reported to solubilize calcium phosphate [56]. A study that applied BA-NH-NH-BA topically in a murine model of uremic itching, considered this compound as a postbiotic [56]. However, this does not comply with the novel definition proposed by the ISAAP panel since a purified microbial metabolite itself cannot be considered a postbiotic [56].

Several studies on postbiotics and kidney disease were not very informative as they studied healthy animals or were too preliminary and did not address in vivo and functional consequences following administration. In aged or adult mice, treatment with probiotics or probiotics and postbiotics mix (Lactobacillus and Bifidobacterium strains and their postbiotics compounds selected for potential antioxidative activity) decreased oxidative stress as assessed by MDA (malondialdehyde) in the kidneys [57]. However, an impact on kidney function was not assessed and whether or not the combination of postbiotics with probiotics added up to the impact of probiotics alone was not formally assessed, although a trend towards a greater impact was observed in the higher dose groups.

Fifteen weeks of a diet supplemented by a postbiotic based on lactic acid bacteria in healthy male rabbits was not associated with differences in kidney function parameters, including serum urea and creatinine [58]. Based on the design, this study should be considered a safety study, as the impact on a disease condition was not assessed.

The postbiotic OM-85 is a standardized lysate of 21 bacterial strains, often found in human airways, that is undergoing clinical trials for diverse respiratory conditions and it has already been authorized in several European countries [59]. The EMA limits its use to the prevention of recurrent respiratory infections [60]. A clinical trial investigating children following the first episode of idiopathic nephrotic syndrome is not yet recruiting (NCT05044169), but plans to enroll 83 patients to whom OM-85 will be administered for 6 months after remission with a primary endpoint of one year relapse-free survival rate. Since nephrotic syndrome relapse is frequently preceded by infections, OM-85 is hypothesized to reduce the incidence of bacterial respiratory infections and, thus, reduce infection-related relapses. Unfortunately, a comparison to placebo was not considered, making the results of the trial difficult to interpret. In cultured epithelial cells, including kidney-derived Vero E6 monkey cells, OM-85 downregulated ACE2 and TMPRSS2 and, as a result, inhibited SARS-CoV-2 cell infection [61]. Whilst these results are promising, the absence of in vivo and clinical studies hampers the translatability and applicability of these observations. Despite the generally weak and/or preliminary data on postbiotics and kidney disease, promising, mainly preclinical, results were reported for postbiotics in hyperoxaluria, AKI, high fat diet-induced kidney disease and hypertension, as discussed below.

## 7. Postbiotics in Hyperoxaluria: *Oxalobacter formigenes* Lysates

In hyperoxaluria, increased oxalate absorption from diet or endogenous oxalate production result in increased urinary oxalate excretion potentially leading to calcium oxalate (CaOx) urolithiasis and CaOx crystal formation in kidney tissue, that can lead to renal calcification and, eventually, to kidney failure and systemic CaOx deposition or oxalosis [62]. CaOx crystals may cause kidney injury, inflammation and tubular obstruction that drive the progressive loss of kidney function, eventually leading to a need of kidney replacement therapy in the most severe cases [63,64,65]. Hyperoxaluria results from either a hepatic oxalate overproduction caused by genetic disorders of glyoxylate metabolism (primary hyperoxaluria) or ingestion of oxalate precursors, or from an elevated intestinal oxalate absorption (secondary hyperoxaluria). Secondary hyperoxaluria is more common and usually milder than primary hyperoxaluria and treatable with diet (low oxalate, calcium-containing diet). However, hyperoxaluria may cause AKI if oxalate ingestion is suddenly excessive (e.g., juicing) especially if this is linked to decreased gut calcium availability (e.g., during fat malabsorption as fat chelates calcium) as gut calcium oxalate crystal are not absorbed but excreted in feces.

Primary hyperoxaluria type 1 (PH1) is a rare genetic disease caused by a deficient liver alanine-glyoxylate transaminase enzyme activity. Being the most severe form of hyperoxaluria, considerable efforts have been made to develop novel therapies. Current treatment options for PH1 are suboptimal. To date, supportive treatments focus on high fluid intake and crystallization inhibitors as well as pyridoxine treatments [66]. Eventual development of kidney failure is, however, associated with oxalosis and premature death. Liver transplantation restores hepatic alanine-glyoxylate transaminase enzyme activity. Novel therapies based on RNA interference (RNAi) can target enzymes upstream and reduce or prevent oxalate production. For this, lumasiran, targeting liver glycolate oxidase (GO) is already approved by EMA and FDA, while nedosiran, targeting liver lactate dehydrogenase A (LDH-A) is currently undergoing RCTs [67].

Probiotics and, more recently, postbiotics have been studied for therapy of preclinical hyperoxaluria and human PH. *O. formigenes* is an anaerobic bacterium found in the gut that might help reducing the risks of developing urinary oxalate stones [68,69]. *O. formigenes* relies solely on oxalate for its growth and is a key oxalate-degrading bacterium that prevents kidney toxicity in animals fed on an oxalate-rich plants diet [69]. Clinical studies suggest an association between the absence of *O. formigenes* in the gut and the development of oxalate stone disease and hyperoxaluria [70,71,72]. Interestingly, treatment with either whole *O. formigenes* to colonize the gut (i.e., probiotics) or encapsulated *O. formigenes* lysates (i.e., postbiotics) reduced urinary oxalate excretion in rats [73]. Artificial or natural colonization of control Sprague-Dawley rats with *O. formigenes* promoted oxalate degradation and there is also evidence for physical interaction with the mucosa initiating colonic oxalate secretion. Urinary oxalate excretion was also decreased. In longer term studies, nephrocalcinosis was reduced [74]. Interestingly, dietary calcium influenced the ability to maintain *O. formigenes* colonization, which was persistent only when dietary calcium was low, i.e., when the amount of available calcium to bind to oxalate was low [73]. This would create a problem for the efficacy of live *O. formigenes* therapy since the potential benefits of *O. formigenes* on oxalate absorption in the gut could be offset by the need to maintain a low calcium diet. The benefits of probiotic could be reproduced by using postbiotic enteric-coated encapsulated *O. formigenes* freeze-dried lysates twice daily for five days that also reduced urinary oxalate excretion by 50% and supported colonic oxalate secretion in hyperoxaluric rats with renal insufficiency [73]. The *O. formigenes* lysate was hypothesized to have both a secretagogue function and an enzymatic degradation effect on luminal oxalate. The gelatin capsules used in the study contained freeze-dried lysate of the *O. formigenes* strain, oxalyl CoA, and thiamine pyrophosphate (8:1:1) and thus fits the current ISAAP definition of a postbiotic. Unfortunately, comparing the results obtained with the probiotic (live *O. formigenes*) and postbiotic (freeze-dead *O. formigenes*) is not possible, since dead bacteria were only tested in rats with renal insufficiency induced by unilateral nephrectomy and not in healthy rats [73]. These results support the idea that the *O. formigenes* postbiotic could contribute to the maintenance of the balance between renal and enteric oxalate [73], however the efficacy of the postbiotic should be confirmed in clinical studies. If efficacious to reduced oxalate load in vivo in humans, postbiotic *O. formigenes* may address several of the issues associated with postbiotic *O. formigenes*: the difficulty to grow and maintain alive a strict anaerobe, the potentially negative impact of calcium-containing diets (a current recommendation to prevent oxalate absorption) on maintaining *O. formigenes* colonization in vivo and the negative impact of antibiotic courses on *O. formigenes* colonization [75,76]. As an additional potential barrier to the success of prebiotic *O. formigenes* therapy, colonization is associated with a more complex microbiota (higher alpha-diversity) and the association of *O. formigenes* with other multiple taxa known to also be stimulated by oxalate in rodent models better differentiated gut microbiota from patients with and live-in individuals without urinary stone disease [75,77,78].These findings suggest that *O. formigenes* may better protect from oxalate associated diseases in conjunction with other components of the microbiota. Eventually, postbiotics may be designed that promote this associated microbiota.

**Figure 3:**
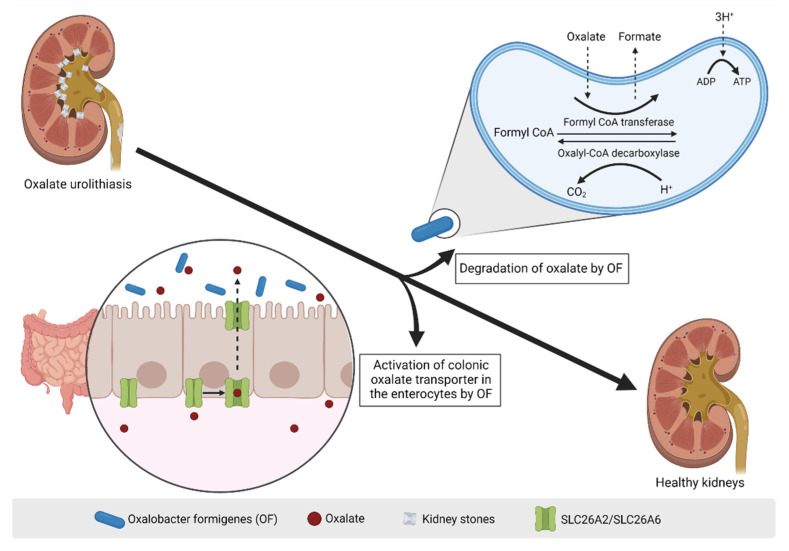
***O. formigenes* and hyperoxaluria.** The activity of the *Oxalobacter formigenes* bacteria resident in the gut has been proposed as a potential mechanism for the treatment of kidney stones via two defined mechanisms: 1. Degradation of oxalate. Oxalate is taken up by the bacteria and transformed into formate via the enzymatic activity of Formyl CoA transferase; 2. Activation of colonic oxalate transporter in the enterocytes. Circulating oxalate is transported by SLC26A2 and SLC26A6 transporters located at the basolateral side of intestinal epithelial cells and gets extracted into the intestinal lumen. Figure created with Biorender.com (license number 9E1540F2).

More recently, *O. formigenes* culture conditioned medium was found to increase oxalate uptake (>2.4 fold) in human intestinal Caco-2-BEE cells when compared to control medium [68]. In contrast, conditioned medium from *Lactobacillus* did not stimulate oxalate uptake. The observed increase in oxalate transport might involve signaling via protein kinase A (PKA), as this was inhibited by H89 and required transport by a 4,4′-diisothiocyanostilbene-2,2′-disulfonic acid (DIDS)-sensitive anionic exchanger. There are two well-known DIDS-sensitive anionic exchangers: SLC26A2 (also known as Sulfate Anion Transporter 1 and diastrophic dysplasia sulfate transporter, DTDST) and SLC26A6 (also known as CFEX and PAT1). SLC26A6 knockout using siRNA led to a 50% decrease of oxalate transport in Caco-2-BEE treated with conditioned medium [68]. These results were not reproduced by others [79], however it should be pointed out that both groups tested different strains of *O. formigenes* that had been previously shown to promote oxalate transport in colonized mouse gut: sheep rumen strain of *Oxalobacter* (OxB, ATTC #35274) [68] and a human strain of *Oxalobacter* (HC-1) [79]. In vivo, in PH1 mice treated with *O. formigenes* conditioned medium, a postbiotic, (rectal administration), the urinary oxalate excretion was significantly reduced (32.5%) and the distal colonic oxalate secretion increased (42%) [68] (Figure 3). Thus, the postbiotic *O. formigenes* OxB, ATTC #35274 conditioned medium modulates oxalate transport in both in vitro cultured human intestinal epithelial cells and in vivo in murine colon. Nonetheless, these observations may not be applicable to other *Oxalobacter* strains. A more recent study observed that the increase in oxalate flux across the colon of mice colonized with live *Oxalobacter* was still observed in mice deficient for the apical oxalate transporters Slc26a6 and Slc26a3/Dra [80], suggesting that other oxalate transporters might be involved as well [79].

Postbiotic preparations of *O. formigenes* should not be confused with Oxabact™, a lyophilized *O. formigenes* formulation that aims at colonizing the gut with live *O. formigenes*. Oxabact™ is a capsule containing lyophilized O. formigenes, strain HC-1 (≥ 10^9^ to  < 5^10^ colony forming units per dose). Since lyophilization does not kill bacteria, Oxabact™ is considered a probiotic. However, Oxabact will be discussed in certain detail as, similar to other prebiotics, the ratio of live/dead bacteria could change during the shelf life, resulting in a variable postbiotics contents whose contribution to any efficacy result remains understudied.

Oxabact™ has been tested in various RCTs: in a phase II, open-label trial aimed at PH1 patients on dialysis, Oxabact™ administration for 24 months decreased plasma oxalate levels and improved or stabilized cardiac function as well as clinical status when compared with placebo [81]. Oxabact™ also improved clinical disease progression in a female infant with severe PH1 [82]. However, placebo-controlled trials were not that successful. The most recent phase III, double-blind, placebo-controlled randomized trial investigated the effectiveness of Oxabact™, orally administered for 1 year, in reducing oxalate levels in PH patients, but failed to find a significant difference in plasma oxalate as compared to placebo (*p*  =  0.06) [83]. Other studies with Oxabact™ also did not observe differences versus placebo, including two randomized, placebo-controlled, double-blind studies assessing urinary oxalate in PH patients treated with Oxabact™ for 24 weeks [84,85]. In this regard, no active Oxabact™ trials are listed in clinicaltrials.gov as of 16 June 2022 and a phase 3 extension study to evaluate the long-term efficacy and safety of Oxabact™ in patients with PH (NCT03938272) was terminated in July 2021 when the parent trial failed to meet the primary endpoint. At the time of termination, no advantage of Oxabact™ was observed for the primary endpoint investigating eGFR. Thus, attempts at human colonization by *O. formigenes* cannot be considered successful so far. Whether this may be the result of the probiotics bioavailability issues or the viability of *O. formigenes* in the formulations, it is currently unclear. Maintaining *O. formigenes* alive proved to be challenging due to its anaerobic need. Its stability could also be a limitation, as this can be affected during both industrial processing and storage. Moreover, the ratio of live/dead bacteria could substantially change during the shelf life, affecting its overall efficacy. Accordingly, to overcome such challenges, a postbiotic approach should be considered by taking into consideration different dosing and administration strategies.

## 8. Diet-Induced Kidney Injury

Postbiotics have been studied in preclinical, but not in clinical diet-induced hypertension and kidney disease. A postbiotic derived from sonicated *Lactobacillus paracasei* isolated from Egyptian cheese was administered to adult Wistar albino male rats fed with a high fat diet for 9 weeks and compared to atorvastatin treated or placebo to assess the impact on weight and lipids [86]. The postbiotic contained several enzymes, including proteases, lipases and antioxidant enzymes (superoxide dismutase, catalase, and glutathione peroxidase) and displayed antibacterial activity against pathogenic bacteria. Both the postbiotic and atorvastatin reduced total serum lipids, serum triglycerides and total serum cholesterol and prevented an increase in body weight. However, the overall body weight of atorvastatin- and postbiotic-treated rats was lower than that of rats fed on a normal diet, which may be a cause for concern. In this regard, there was evidence for atorvastatin toxicity such as increased liver enzymes and bilirubin levels, whereas this was not observed for the postbiotic treatment. A high fat diet results in kidney disease characterized by an increase in serum creatinine, uric acid and urea levels, and, surprisingly, this was exacerbated by atorvastatin. In postbiotic-treated rats instead, serum creatinine, uric acid and urea levels remained in the normal range and were lower than in high fat diet rats with or without atorvastatin treatment [86]. Although the molecular mechanisms or underlying kidney pathohistology were not explored, the postbiotic treatment appeared to have a protective effect from a high fat diet-induced kidney dysfunction.

A potential postbiotic that has also been studied in a preclinical diet-induced hypertension and kidney injury model is GABA-salt. For preclinical studies, GABA-salt was prepared by culturing Lactobacillus brevis BJ20 for 24 h and then further fermented with 40% (*w/w*) refined seawater salt for 6 h and then filtered and spray dried to yield GABA-salt. GABA-salt contained 0.76% ± 0.01% GABA, as well as other unmeasured postbiotics components [87]. Based on the information provided in the manuscript, no purification step appears to have been performed to eliminate other bacterial metabolites. Oral GABA-salt and salt treatment were compared in a murine model of hypertension induced by a high salt and high cholesterol diet. Replacing salt for GABA-salt attenuated the diet-induced increase in serum creatinine and urea, blood pressure, intima-media thickness, and other changes in aorta, such as M1 polarization (CD86 expression), cell death (TUNEL staining) and TNF-α and inducible nitric oxide synthase (NOS) levels. Furthermore, GABA-salt induced changes consistent with the in vivo observations in cultured macrophages, endothelial cells, and vascular smooth muscle cells. While the authors hypothesized that any impact of GABA-salt was due to the GABA content, the GABA-salt preparation would be expected to contain other bacterial products, i.e., to be a postbiotic, and from the experiments performed, it remains unclear which individual or combination of postbiotic components was responsible for the observed effect. Indeed, it was not tested whether GABA itself added to control salt resulted in the same effects as the postbiotic GABA-salt.

GABA-salt studies followed prior description of a fermented milk containing GABA by using *Lactobacillus casei* strain Shirota and *Lactococcus lactis* YIT 2027 that lowered blood pressure in humans and rats [88,89]. The *Lb. casei* strain hydrolyzes milk protein into glutamic acid, and the *Lc. lactis* converts glutamic acid into GABA (GABA 10–12 mg/dL). However, from the manuscripts, it is unclear whether the fermented milk contained probiotics (i.e., live bacteria) and/or postbiotics (i.e., dead bacteria and their products) [89]. The human study was a pilot study in which 39 mildly hypertensive patients received daily GABA fermented milk or placebo (non-fermented milk) for 12-weeks. Systolic blood pressure decreased by 17.4 ± 4.3 mmHg in the intervention group and this was reported to be statistically significantly different from the placebo group, in which blood pressure also decreased [89]. In SHR hypertensive rats, a diet containing freeze-dried GABA fermented milk (100 g/kg, final concentration of GABA 0.1 g/kg) prevented the progressive increase in systolic blood pressure observed between 7 and 10 weeks of age [88].

## 9. Postbiotics in Nephrotoxic AKI

Postbiotics have been studied in preclinical, but not in clinical AKI. In preclinical studies, AKI was induced by cisplatin administration to mice and the severity of injury was magnified by the oral administration of salt from 1 h prior to cisplatin up to 48 h post induction of AKI, with a 72 h readout [90]. GABA-salt was prepared as for the hypertension studies [87] with slight modifications involving further fermentation of Lactobacillus. plantarum BJ21, to yield GABA-salt, and two other compounds that were termed lacto-GABA-salt and postbiotic-GABA-salt. The manuscript does not provide further details on the differential processes performed/used to generate the three compounds or their full composition other than indicating that they contained 93, 112, and 97 mg/g GABA, and that 108, 175 and 109 mg/g were neither sodium chloride nor GABA, respectively. The sodium chloride content of the parent seawater salt was not reported. The results are not very clearly expressed, but it appears that salt administration increased the severity of cisplatin-induced AKI as assessed by serum creatinine and urea, histological injury and expression of inflammatory mediators, while the different GABA-salt was protective, the results for lacto-GABA-salt and postbiotic-GABA-salt being unclear [90].

## 10. A Roadmap towards Postbiotic Therapy for Kidney Disease

**Figure 4:**
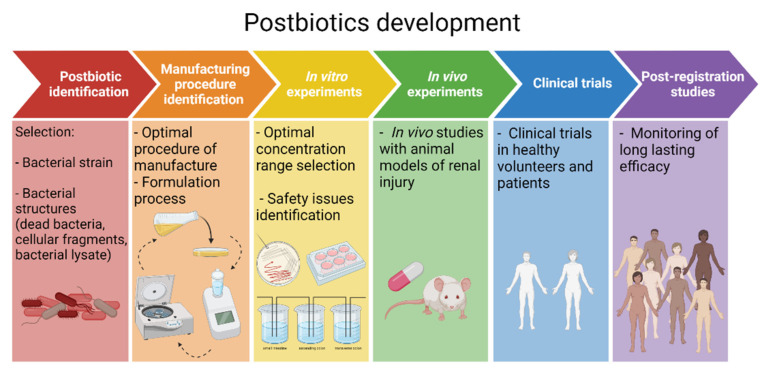
**A roadmap towards postbiotic therapy for kidney disease**. Roadmap displaying the postbiotic development steps from initial postbiotics identification up to the final post-registration studies. In the specific case of kidney disease, initial candidates for this roadmap include *O. formigenes* postbiotics for enteric hyperoxaluria-related kidney injury and urolithiasis. Figure created with Biorender.com (license number 9E1540F2).

The ultimate aim of preclinical research is to develop products that could improve the diagnosis and treatment of diseases. Postbiotics are not well-known and postbiotics research is still in its early stages and has not yet led to products for clinical use. Indeed, as recently as July 2022 postbiotics were still erroneously defined as therapeutic strategies that target downstream signaling pathways of microbiome [91]. We suggest a roadmap for the clinical development of postbiotics in the field of kidney disease (Figure 4). First, it would be necessary to identify the key bacterial strains that play an important role in the development and progression of kidney disease and define the mechanisms underlying their beneficial function as it might be that not all the bacterial components might be involved. Recognizing which fragments have a functional role and which do not would be of extreme importance in the development of postbiotics. A postbiotic approach would overcome limitations faced with probiotics related products, this mainly being their instability. Furthermore, it is necessary to characterize the optimal procedures of manufacturing, formulation and administration, and in vitro experiments are required to establish the optimal concentration range and to identify early any safety issues. In vitro studies should address any positive or negative impact of the postbiotic on cultured mammal cells, including human gut, kidney and immune cells, as well as on complex bacterial communities, using technologies such as the Dynamic Gastrointestinal Simulator (SIMGI^®^) [92]. Tubular cells are the key kidney cell types to study, given that they represent most of the kidney mass and have an array of transporters and receptors, although glomerular and endothelial cells may also be of interest. Subsequently, in vivo experiments with animal models of kidney injury are required. Given the short timelines, preclinical AKI models may be of interest. Moreover, some of them result in features of CKD within a reasonable time frame (the so-called AKI-to-CKD transition), so insight into chronicity may be gained from these studies. However, no AKI therapy identified in preclinical models has ever reached routine clinical use, pointing to potentially large differences in pathophysiology and/or timing of the intervention between preclinical models and human disease. In this regard, models representing clinical situations in which the inventio may be applied prophylactically in the clinic may offer advantages (e.g., kidney ischemia during surgery, exposure to nephrotoxic chemotherapeutic agents). In order to be effective, postbiotics should be prepared in such a way that it makes them resistant to degradation whilst reaching the targeted segment of the gastrointestinal tract. Manufacturing procedures should ensure low variability of the finished product and scalability to produce large batches. In this regard, several postbiotics possibilities are available, ranging from bacterial lysates to conditioned cultured medium to extracellular vesicles as recently demonstrated for *O. formigenes* [93]. Moreover, more complex postbiotcs consisting of diverse bacteria encoding oxalate degradation pathways may be explored [72,94]. Finally, RCTs in patients with kidney diseases should be planned. Short-term trials should establish their safety in humans and explore biomarkers of their biological activity (e.g., oxaluria in patients with hyperoxaluria). Larger efficacy trials focusing on broader endpoints will be more difficult to design and fund but are required to gather the dataset necessary for approval by the medicine’s regulatory agencies. In this regard, PH1 is a rare disease which has severe, potentially life-threatening complications and treatment of PH1 represents both an unmet clinical need and an attractive target from the human and regulatory point of view. However, being a severe condition in which oxalic acid is produced endogenously, PH1 may be less amenable to interventions acting at the level of the gut lumen. A shortcut to postbiotics as a drug treatment would be to consider them as nutritional supplements or medical nutrition. This would increase the feasibility factor for clinical studies, but would decrease the trustworthiness of any claim of health benefit. *O. formigenes* postbiotics may be suitable for assessment as nutritional supplements in hyperoxaluria of enteric origin. This condition is more common and less severe than PH1, facilitating clinical trials or nutritional research; the via of entry of oxalic acid in the body is by ingestion, similar to the postbiotics, which may increase efficacy and finally; there is a subset of health-conscious but ill-advised persons with hyperoxaluria causing kidney injury resulting from fashionable trends or diets (e.g., juicing) who would may also be prone to taking supplements such as *O. formigenes* postbiotics, especially if this would allow then to pursue their diets more safely. Another potential scenario of *O. formigenes* postbiotics use if prevention of antibiotic-associated urolithiasis, especially if long-term antibiotics are needed, as while on antibiotics, attempts at colonization by *O. formigenes* probiotics are expected to be futile [95]. Once on the market, post-registration studies will monitor the long-lasting safety and efficacy effects. In the case of postbiotics for enteric hyperoxaluria, the whole process from research to marketing and post marketing experience may allow to learn enough about the intervention to re-design it for more severe conditions such as PH1.

## Figures and Tables

**Table 1:** Intervention studies of postbiotics in adult humans identified by Salminen et al. [5]. Although no specific date for the search was provided, references up to 2020 were cited.

Intervention Group	Postbiotic	Reference
Helicobacter pylori	Inactivated culture of *Lactobacillus*	[24]
Irritable bowel syndrome and diarrhea	Lacteol, inactivated *L. acidophilus LB* plus fermented culture medium	[25]
Irritable bowel syndrome	Non-viable, heat-inactivated *Bifidobacterium bifidum MIMBb75*	[26]
Chronic diarrhea	Heat-killed *L. acidophilus LB* (Lacteol Fort)	[27]
Obstructive jaundice	Inactivated *Lactiplantibacillus plantarum*	[28]
Stress response in undergraduate medical students	Heat-inactivated *L. gasseri* strain CP2305	[29]
Chronic responses in medical students	Heat-inactivated *L. gasseri* strain CP2305	[30]
Response (endocrine, inflammation, performance) during self-defense training in soldiers	Inactivated *Bacillus coagulans*	[31]
Latent tuberculosis	Heat-killed *Mycobacterium manresensis*	[32]
Asthma	Inactivated *Mycobacterium phlei*	[33]
Chronic obstructive pulmonary disease	Inactivated, non-typable *H. influenzae*	[34]
Bacterial colonization of nose and throat	Lysate containing *S. aureus, Streptococcus mitis, S. pyogenes, S. pneumoniae, K. pneumoniae, M. catarrhalis, H. influenzae*	[35]
Chronic obstructive pulmonary disease	Lyophilized bacterial fragments derived from *S. aureus, Streptococcus viridans, S. Pneumoniae, Klebsiella ozaenae, M. catarrhalis, H. influenzae*	[36]
Recurrent respiratory tract infectious	*Lantigen B*, suspension of bacterial antigens obtained from *S. pneumoniae type 3, S. pyrogenes group A, B. catarrhalis, S. aureus, H. influenzae type B* and *K. pneumoniae*	[37]
Cancer and leukopenia following chemotherapy	DEODAN, lysozyme lysates of *Lactobacillus bulgaricus*	[38]

**Table 2:** Intervention studies of postbiotics in pediatric patients identified by Salminen et al. [5]. Although no specific date for the search was provided, references up to 2020 were cited.

Intervention Group	Postbiotic	References
Fermented formula (healthy infants)	Fermented formula with *BB C50* and *ST065*	[39,40,41,42,43]
Fermented formula in preterm infants	Heat-inactivated fermented formula with *BB C50* and *ST 065*	[44]
Acute gastroenteritis	Heat-killed *Lactobacillus LB*	[45,46,47,48]
Prevention of common infectious diseases	Heat-inactivated *L. paracasei CBA L74* or *L. acidophilus*	[49,50,51]
Atopic eczema and cow’s milk allergy	Live or heat-inactivated *L. rhamnosus*	[52]
Allergic rhinitis	Live or heat-killed *L. paracasei 33*	[53]
Lactose malabsorption	Killed and live *Lactobacillus helveticus R-52* and *L. rhamnosus R-11*	[54]

## Data Availability

Not applicable.

## Supplementary Materials

The following supporting information can be downloaded at: https://www.mdpi.com/article/10.3390/toxins14090623/s1, Search strategy and results.
