# Supplementary material for: Postbiotics and Kidney Disease
Source: Toxins (Basel). 2022 Sep 6;14(9):623. doi: 10.3390/toxins14090623 (PMC9501217; doi:10.3390/toxins14090623)
Supplement: Supplementary file 1 [file toxins-14-00623-s001.zip › toxins-1862117-supplementary.pdf]

Supplementary Materials: Postbiotics and Kidney Disease

Chiara Favero, Laura Giordano, Silvia Maria Mihaila, Roosalinde Masereeuw, Alberto Ortiz and Maria Dolores Sanchez-Niño

Citation: Favero, C.; Giordano, L.; Mihaila, SM.; Masereeuw, R.; Ortiz, A.; Sanchez-Niño, M.D. Postbiotics and Kidney Diseases. *Toxins* **2022**, *14*, 623. https://doi.org/10.3390/toxins14090623

Received: 27 July 2022
Accepted: 1 September 2022
Published: 6 September 2022

Publisher's Note: MDPI stays neutral with regard to jurisdictional claims in published maps and institutional affiliations.

Copyright: © 2022 by the authors. Submitted for possible open access publication under the terms and conditions of the Creative Commons Attribution (CC BY) license (https://creativecommons.org/licenses/by/4.0/).

Search strategy and results

Different search strategies to identify original manuscripts exploring the role of postbiotics in preclinical or clinical studies of kidney disease or conditions related to kidney disease (e.g. hypertension) were performed in May 2022:

The first strategy searching for "postbiotic" AND "kidney", yielded a total of 13 manuscripts: in 8 the concept of postbiotic was considered only broadly [1–8] and were thus were excluded. The remaining 5 were included and discussed in this present manuscript [9–13]. Among the included studies, one manuscript incorrectly used the term postbiotic by referring to a purified microbial metabolite, i.e. N-[2-(2-Butyrylamino-ethoxy)-ethyl]-butyramide (BA-NH-NH-BA) [13].

A search for "Hypertension" AND "Postbiotic" found 11 results: most of which were reviews [1,4,7,14–18]. Two of them were designs of clinical trials, one did not use a postbiotic intervention and the other considered SCFAs as postbiotics in the introduction section, although the intervention was a prebiotic[19,20]. A further clinical trial, which was unrelated to hypertension, also considered butyrate as a postbiotic [21]. Thus, no hits from this search could be used in the present review.

Finally, a PubMed search using ("kidney" OR "hypertension") and the individual postbiotics cited by Salminen et al ("deodan" OR "lyophilized heat-killed bacteria" OR "lyophilized bacterial fragments" OR "lantigen b" OR "bacterial lysate" OR "heat-killed l. paracasei" OR "lantigen" [22] yielded 11 hits of which 10 were excluded: one targeted respiratory infections [23]; two were not related to kidney disease or hypertension [24,25]; and the others were *in vitro* experiments or studies in which the "bacterial lysate" was only cited for other purposes [26–31]. Only one study used a bacterial lysate in cultured kidney cells and is discussed in the present review [32].

References

1. Muralitharan, R.R.; Jama, H.A.; Xie, L.; Peh, A.; Snelson, M.; Marques, F.Z. Microbial Peer Pressure: The Role of the Gut Microbiota in Hypertension and Its Complications. *Hypertension* **2020**, *76*, 1674–1687. https://doi.org/10.1161/HYPERTENSIONAHA.120.14473.
2. Wernimont, S.M.; Radosevich, J.; Jackson, M.I.; Ephraim, E.; Badri, D.V.; MacLeay, J.M.; Jewell, D.E.; Suchodolski, J.S. The Effects of Nutrition on the Gastrointestinal Microbiome of Cats and Dogs: Impact on Health and Disease. *Front Microbiol* **2020**, *11*, 1266, doi:10.3389/fmicb.2020.01266.
3. Andrade-Oliveira, V.; Foresto-Neto, O.; Watanabe, I.K.M.; Zatz, R.; Câmara, N.O.S. Inflammation in Renal Diseases: New and Old Players. *Front Pharmacol* **2019**, *10*, 1192, doi:10.3389/fphar.2019.01192.
4. Noce, A.; Marrone, G.; Di Daniele, F.; Ottaviani, E.; Wilson Jones, G.; Bernini, R.; Romani, A.; Rovella, V. Impact of Gut Microbiota Composition on Onset and Progression of Chronic Non-Communicable Diseases. *Nutrients* **2019**, *11*, E1073, doi:10.3390/nu11051073.

5. Tsai, Y.-L.; Lin, T.-L.; Chang, C.-J.; Wu, T.-R.; Lai, W.-F.; Lu, C.-C.; Lai, H.-C. Probiotics, Prebiotics and Amelioration of Diseases. *J Biomed Sci* **2019**, *26*, 3, doi:10.1186/s12929-018-0493-6.
6. Cosola, C.; Rocchetti, M.T.; Cupisti, A.; Gesualdo, L. Microbiota Metabolites: Pivotal Players of Cardiovascular Damage in Chronic Kidney Disease. *Pharmacol Res* **2018**, *130*, 132–142, doi:10.1016/j.phrs.2018.03.003.
7. Hsu, C.-N.; Tain, Y.-L. Chronic Kidney Disease and Gut Microbiota: What Is Their Connection in Early Life? *Int J Mol Sci* **2022**, *23*, 3954, doi:10.3390/ijms23073954.
8. Chou, Y.-T.; Kan, W.-C.; Shiao, C.-C. Acute Kidney Injury and Gut Dysbiosis: A Narrative Review Focus on Pathophysiology and Treatment. Int J Mol *Sci* **2022**, *23*, 3658, doi:10.3390/ijms23073658.
9. Díaz Cano, J.V.; Argente, M.-J.; García, M.-L. Effect of Postbiotic Based on Lactic Acid Bacteria on Semen Quality and Health of Male Rabbits. *Animals* **2021**, *11*, 1007. https://doi.org/10.3390/ani11041007.
10. Osman, A.; El-Gazzar, N.; Almanaa, T.N.; El-Hadary, A.; Sitohy, M. Lipolytic Postbiotic from Lactobacillus Paracasei Manages Metabolic Syndrome in Albino Wistar Rats. *Molecules* **2021**, *26*, 472. https://doi.org/10.3390/molecules26020472.
11. Lin, W.-Y.; Lin, J.-H.; Kuo, Y.-W.; Chiang, P.-F.R.; Ho, H.-H. Probiotics and Their Metabolites Reduce Oxidative Stress in Middle-Aged Mice. Curr. *Microbiol*. **2022**, *79*, 104. https://doi.org/10.1007/s00284-022-02783-y.
12. Lee, H.; Ji, S.Y.; Hwangbo, H.; Kim, M.Y.; Kim, D.H.; Park, B.S.; Park, J.-H.; Lee, B.-J.; Kim, G.-Y.; Jeon, Y.-J.; et al. Protective Effect of Gamma Aminobutyric Acid against Aggravation of Renal Injury Caused by High Salt Intake in Cisplatin-Induced Nephrotoxicity. Int. J. Mol. *Sci*. **2022**, *23*, 502. https://doi.org/10.3390/ijms23010502.
13. Keshari, S.; Wang, Y.; Herr, D.R.; Wang, S.-M.; Yang, W.-C.; Chuang, T.-H.; Chen, C.-L.; Huang, C.-M. Skin Cutibacterium Acnes Mediates Fermentation to Suppress the Calcium Phosphate-Induced Itching: A Butyric Acid Derivative with Potential for Uremic Pruritus. J. Clin. *Med*. **2020**, *9*, 312. https://doi.org/10.3390/jcm9020312.
14. Mishima, E.; Abe, T. Role of the Microbiota in Hypertension and Antihypertensive Drug Metabolism. *Hypertens Res* **2022**, *45*, 246–253, doi:10.1038/s41440-021-00804-0.
15. Avolio, E.; Gualtieri, P.; Romano, L.; Pecorella, C.; Ferraro, S.; Palma, G.; Di Renzo, L.; De Lorenzo, A. Obesity and Body Composition in Man and Woman: Associated Diseases and the New Role of Gut Microbiota. *Curr Med Chem* **2020**, *27*, 216–229, doi:10.2174/0929867326666190326113607.
16. Hsu, C.-N.; Hou, C.-Y.; Hsu, W.-H.; Tain, Y.-L. Cardiovascular Diseases of Developmental Origins: Preventive Aspects of Gut Microbiota-Targeted Therapy. *Nutrients* **2021**, *13*, 2290, doi:10.3390/nu13072290.
17. Zhang, L.; Ko, C.-Y.; Zeng, Y.-M. Immunoregulatory Effect of Short-Chain Fatty Acids from Gut Microbiota on Obstructive Sleep Apnea-Associated Hypertension. *Nat Sci Sleep* **2022**, *14*, 393–405, doi:10.2147/NSS.S354742.
18. Kim, T.T.; Parajuli, N.; Sung, M.M.; Bairwa, S.C.; Levasseur, J.; Soltys, C.-L.M.; Wishart, D.S.; Madsen, K.; Schertzer, J.D.; Dyck, J.R.B. Fecal Transplant from Resveratrol-Fed Donors Improves Glycaemia and Cardiovascular Features of the Metabolic Syndrome in Mice. *Am J Physiol Endocrinol Metab* **2018**, *315*, E511–E519, doi:10.1152/ajpendo.00471.2017.
19. Rhys-Jones, D.; Climie, R.E.; Gill, P.A.; Jama, H.A.; Head, G.A.; Gibson, P.R.; Kaye, D.M.; Muir, J.G.; Marques, F.Z. Microbial Interventions to Control and Reduce Blood Pressure in Australia (MICRoBIA): Rationale and Design of a Double-Blinded Randomised Cross-over Placebo Controlled Trial. *Trials* **2021**, *22*, 496. https://doi.org/10.1186/s13063-021-05468-2.
20. Fan, L.; Ren, J.; Chen, Y.; Wang, Y.; Guo, Z.; Bu, P.; Yang, J.; Ma, W.; Zhu, B.; Zhao, Y.; et al. Effect of Fecal Microbiota Transplantation on Primary Hypertension and the Underlying Mechanism of Gut Microbiome Restoration: Protocol of a Randomized, Blinded, Placebo-Controlled Study. *Trials* **2022**, *23*, 178, doi:10.1186/s13063-022-06086-2.
21. Lewandowski, K.; Kaniewska, M.; Karłowicz, K.; Rosołowski, M.; Rydzewska, G. The Effectiveness of Microencapsulated Sodium Butyrate at Reducing Symptoms in Patients with Irritable Bowel Syndrome. *Prz Gastroenterol* **2022**, *17*, 28–34, doi:10.5114/pg.2021.112681.
22. Salminen, S.; Collado, M.C.; Endo, A.; Hill, C.; Lebeer, S.; Quigley, E.M.M.; Sanders, M.E.; Shamir, R.; Swann, J.R.; Szajewska, H.; et al. The International Scientific Association of Probiotics and Prebiotics (ISAPP) Consensus Statement on the Definition and Scope of Postbiotics. *Nat Rev Gastroenterol Hepatol* **2021**, *18*, 649–667, doi:10.1038/s41575-021-00440-6.
23. Pozzi, E.; Serra, C. Efficacy of Lantigen B in the Prevention of Bacterial Respiratory Infections. *Monaldi Arch Chest Dis* **2004**, *61*, 19–27.
24. Thwaite, R.; Ji, J.; Torrealba, D.; Coll, J.; Sabés, M.; Villaverde, A.; Roher, N. Protein Nanoparticles Made of Recombinant Viral Antigens: A Promising Biomaterial for Oral Delivery of Fish Prophylactics. *Front Immunol* **2018**, *9*, 1652, doi:10.3389/fimmu.2018.01652.

25. Biswas, G.; Korenaga, H.; Nagamine, R.; Takayama, H.; Kawahara, S.; Takeda, S.; Kikuchi, Y.; Dashnyam, B.; Kono, T.; Sakai, M. Cytokine Responses in the Japanese Pufferfish (Takifugu Rubripes) Head Kidney Cells Induced with Heat-Killed Probiotics Isolated from the Mongolian Dairy Products. *Fish Shellfish Immunol* **2013**, *34*, 1170–1177, doi:10.1016/j.fsi.2013.01.024.
26. Morosanova, M.A.; Plotnikov, E.Y.; Zorova, L.D.; Pevzner, I.B.; Popkov, V.A.; Silachev, D.N.; Jankauskas, S.S.; Babenko, V.A.; Zorov, D.B. Mechanisms of Inflammatory Injury of Renal Tubular Cells in a Cellular Model of Pyelonephritis. *Biochemistry (Mosc)* **2016**, *81*, 1240–1250, doi:10.1134/S000629791611002X.
27. O'Dell, W.D.; Stevens, A.R. Quantitative Growth of Naegleria in Axenic Culture. *Appl Microbiol* **1973**, *25*, 621–627, doi:10.1128/am.25.4.621-627.1973.
28. Cetani, F.; Costagliola, S.; Tonacchera, M.; Panneels, V.; Vassart, G.; Ludgate, M. The Thyroperoxidase Doublet Is Not Produced by Alternative Splicing. *Mol Cell Endocrinol* **1995**, *115*, 125–132, doi:10.1016/0303-7207(95)03680-6.
29. Chicherin, I.Y.; Pogorelky, I.P.; Lundovskikh, I.A.; Darmov, I.V.; Gorshkov, A.S.; Shabalina, M.R. [INTESTINAL FAILURE AND YERSINIA PSEUDOTUBERCULOSIS TRANSLOCATION IN THE DEVELOPMENTOF EXPERIMENTAL GENERALIZED INFECTION]. *Eksp Klin Gastroenterol* **2016**, 24–31.
30. Gandomkar, M.; Najafi, R.; Shafiei, M.; Mazidi, M.; Goudarzi, M.; Mirfallah, S.H.; Ebrahimi, F.; Heydarpor, H.R.; Abdie, N. Clinical Evaluation of Antimicrobial Peptide [(99m)Tc/Tricine/HYNIC(0)]Ubiquicidin 29-41 as a Human-Specific Infection Imaging Agent. *Nucl Med Biol* **2009**, *36*, 199–205, doi:10.1016/j.nucmedbio.2008.11.003.
31. Rosaschino, F.; Cattaneo, L. Strategies for Optimizing Compliance of Paediatric Patients for Seasonal Antibacterial Vaccination with Sublingually Administered Polyvalent Mechanical Bacterial Lysates (PMBL). *Acta Biomed* **2004**, *75*, 171–178.
32. Pivniouk, V.; Pivniouk, O.; DeVries, A.; Uhrlaub, J.L.; Michael, A.; Pivniouk, D.; VanLinden, S.R.; Conway, M.Y.; Hahn, S.; Malone, S.P.; et al. The OM-85 Bacterial Lysate Inhibits SARS-CoV-2 Infection of Epithelial Cells by Downregulating SARS-CoV-2 Receptor Expression. *J. Allergy Clin. Immunol.* **2022**, *149*, 923–933.e6. https://doi.org/10.1016/j.jaci.2021.11.019.
